# Supplementary material for: Multi-cohort, cross-species urinary proteomics reveals signatures of LRRK2 dysfunction in Parkinson’s disease
Source: Mol Syst Biol. 2026 Jan 29;22(5):712–37. doi: 10.1038/s44320-026-00190-0 (PMC13144513; doi:10.1038/s44320-026-00190-0)
Supplement: Supplementary file 1 — Appendix [file 44320_2026_190_MOESM1_ESM.pdf]

Multi-cohort, cross-species urinary proteomics reveals signatures of LRRK2 dysfunction in Parkinson's disease

Duc Tung Vu[1], William Sibran[2], Andreas Metousis[1], Laurine Vandewynckel[2], Basak Eraslan[3], Liesel Goveas[2], Ericka CM Itang[1], Claire Deldycke[2], Adriana Figueroa-Garcia[2], Réginald Lefèbvre[2], Johannes Bruno Müller-Reif[1], Sebastian Virreira Winter[4], Marie-Christine Chartier-Harlin[2,*], Jean-Marc Taymans[2,*], Matthias Mann[1,*] and Ozge Karayel[1,5,*]

[1]Department of Proteomics and Signal Transduction Max Planck Institute of Biochemistry Martinsried Germany
[2]Univ. Lille, Inserm, CHU Lille, UMR-S 1172—LilNCog—Lille Neuroscience & Cognition, F-59000, Lille, France
[3]ML and Bioinformatics, Arc Institute, Palo Alto, CA 94304, USA.
[4]ions.bio GmbH, Am Klopferspitz 19, 82152 Planegg, Germany
[5]Current addresses: Genentech Inc, South San Francisco, CA 94080, USA.
[*]Correspondence: oezgekarayel@gmail.com, mmann@biochem.mpg.de, jean-marc.taymans@inserm.fr and marie-christine.chartier-harlin@inserm.fr

Table of Contents

Appendix Figure S1 Quality control plots of PPMI cohort 3

Appendix Figure S2 Analysis of network proteins 4

Appendix Figure S3 Stratified K-Fold for Machine Learning 5

Appendix Figure S4 Prediction of *LRRK2*^{G2019S} status 6

Appendix Figure S5 Completeness and importance of model features 7

Appendix Figure S6 Cross-cohort prediction of *LRRK2* mutation status 8

Appendix Figure S7 Genetic risk variants share *LRRK2*^{G2019S} like signature 10

Appendix Figure S8 Machine learning-based classification of *PD* 11

Appendix Figure S9 Machine learning-based prediction of *LRRK2*^{G2019S} status in PD patients 12

Appendix Figure S10 Batch correction of combined PPMI, LCC and Columbia data 14

Appendix Figure S11 Peripheral effects of Lrrk2 deficiency, hyperactivation, and inhibition in rat urine.15

Appendix Figure S12 Effects of Lrrk2 deficiency, hyperactivation, and inhibition in rat kidney, lung, brain and urine 16

Appendix Figure S13 Correlation analysis in rat urine, kidney, lung and brain 17

Appendix Figure S14 Model performance of significant rat genes in BAC-*LRRK2*^{G2019S} rats for human *LRRK2*^{G2019S} classification 19

Appendix Figure S15 Targeted proteomic analyses of phosphorylated and unphosphorylated LRRK2 and Rab10 peptides in the PPMI urine dataset. 20

Appendix Figure S16: Distribution of genetic mutations across disease states 22

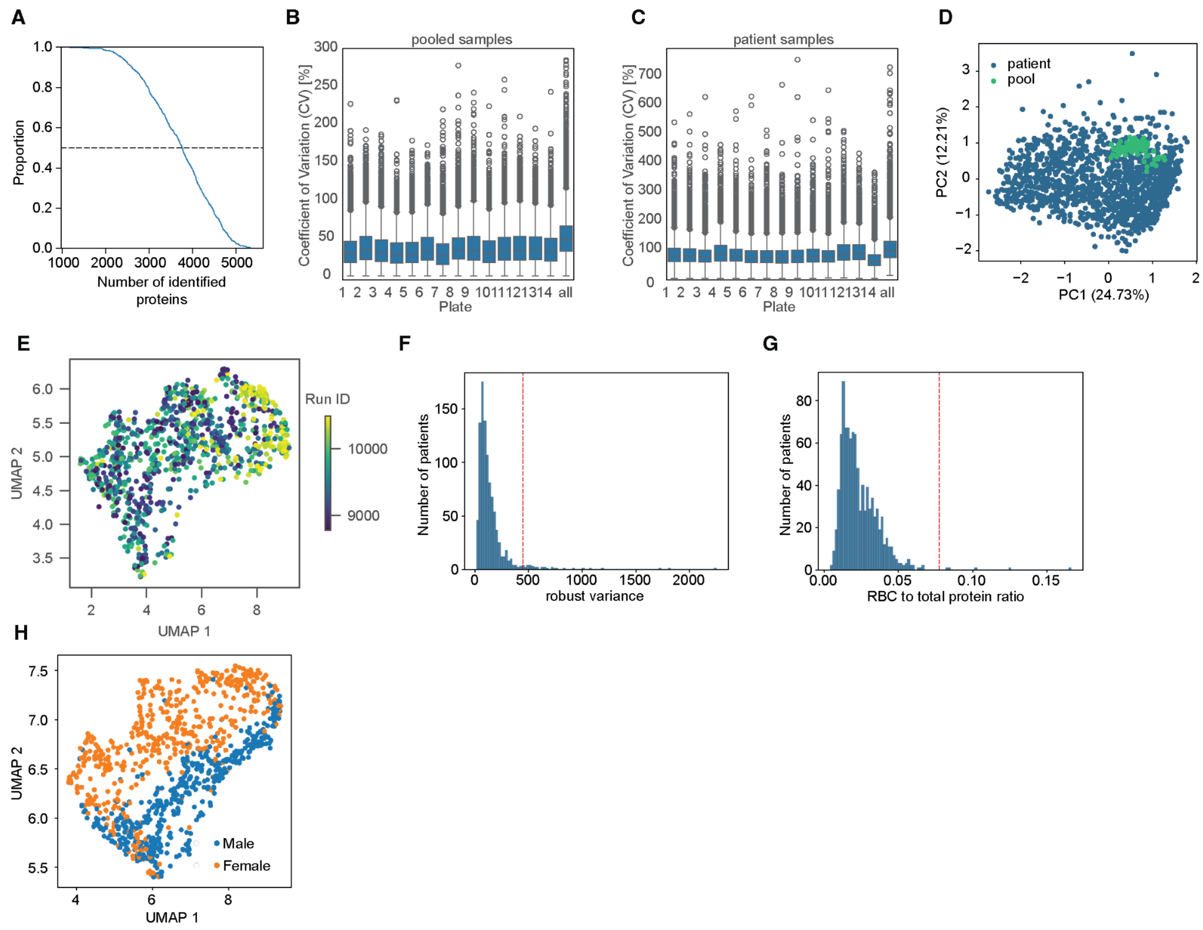

Appendix Figure S1 Quality control plots of PPMI cohort

A. Cumulative distribution plot of numbers of identified proteins in the PPMI urine dataset. Black dotted line represents the median.

B. Coefficient of variation (CV) distribution in % for each plate containing the pooled samples. The last boxplot annotated as "all" represents the overall CV across all samples independent of plate.

C. Same as (A) but for patient samples.

D. Principal component 1 (PC1) and PC2 projection of patient and pooled samples. Annotated value in percent represents the eigenvalue of each principal component.

E. UMAP reduced data with color coding corresponding to Run ID.

F. Distribution of patient samples against numbers of robust variance. The red dotted line represents the outlier cutoff. Patient samples above the cutoff were removed from analysis.

G. Same as (E) but for the red blood cell (RBC) to total protein ratio.

H. UMAP reduced data with color coding corresponding to different sex.

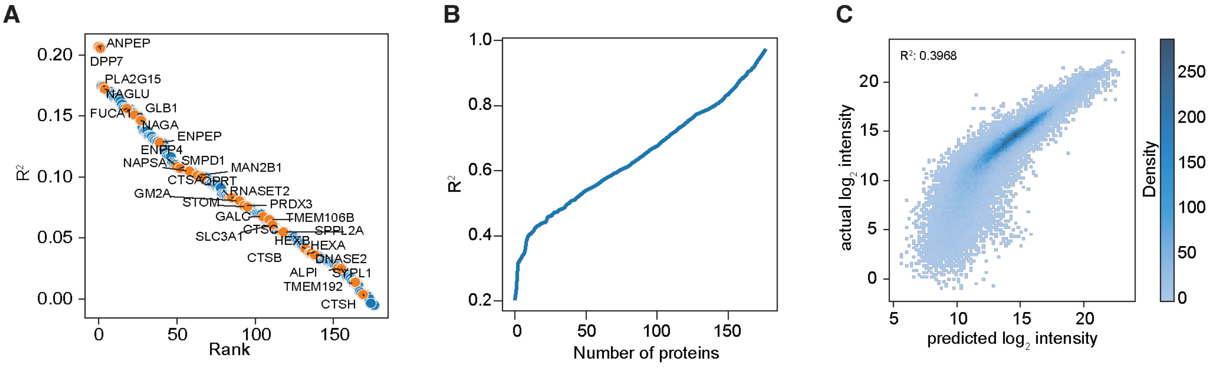

Appendix Figure S2 Analysis of network proteins

A. Ranking of n=177 network proteins based on individual R^2 values, with proteins significantly altered by *LRRK2*^{G2019S} in both LCC and Columbia cohorts annotated.
B. Ridge regression performance (R^2) against number of proteins used as input for $\log_2$ intensity prediction. Proteins were incrementally added to the model by decreasing individual R^2.
C. 2D histogram of predicted versus actual $\log_2$ intensity for the n=177 network proteins.

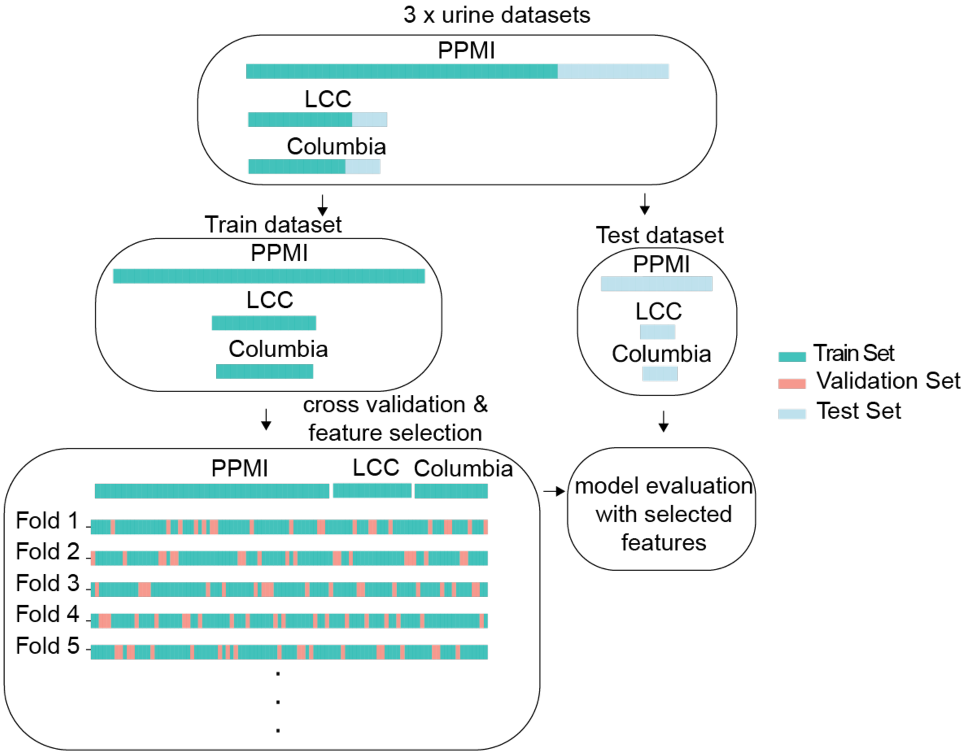

Appendix Figure S3 Stratified K-Fold for Machine Learning

Stratified K-fold strategy for model training and evaluation.

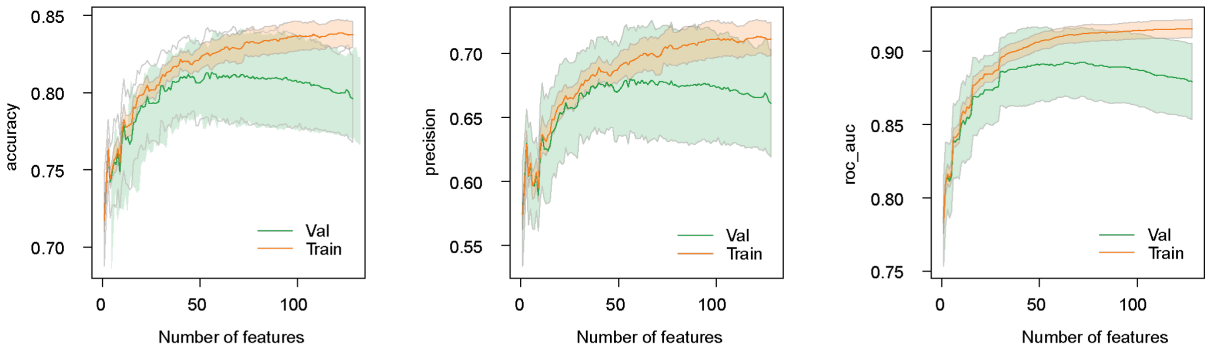

Appendix Figure S4 Prediction of *LRRK2*^{G2019S} status

Evaluation of overfitting with increasing numbers of features as input using accuracy (left), precision (middle), and ROC AUC (right) for prediction of *LRRK2* mutation status. The evaluation was done on the combined (PPMI, LCC & Columbia) urine training and validation data. The shaded areas indicate ±1 standard deviation.

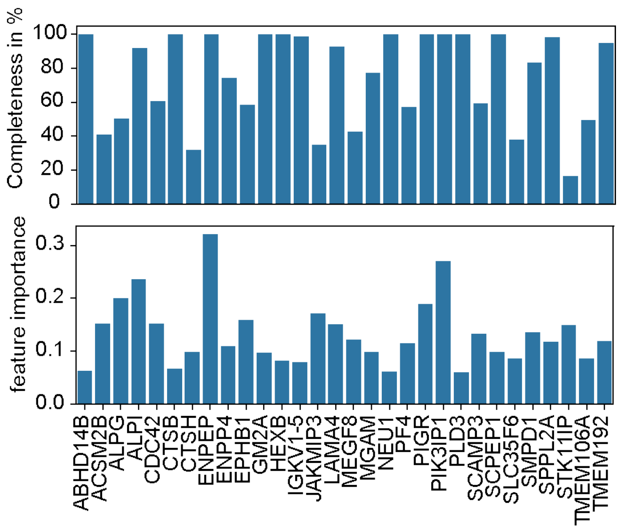

Appendix Figure S5 Completeness and importance of model features

Completeness in % and importance of selected features (30 in total) for *LRRK2*^{G2019S} prediction. Feature importance is the absolute coefficient of the SVM. Completeness is the percentage of identification across patients.

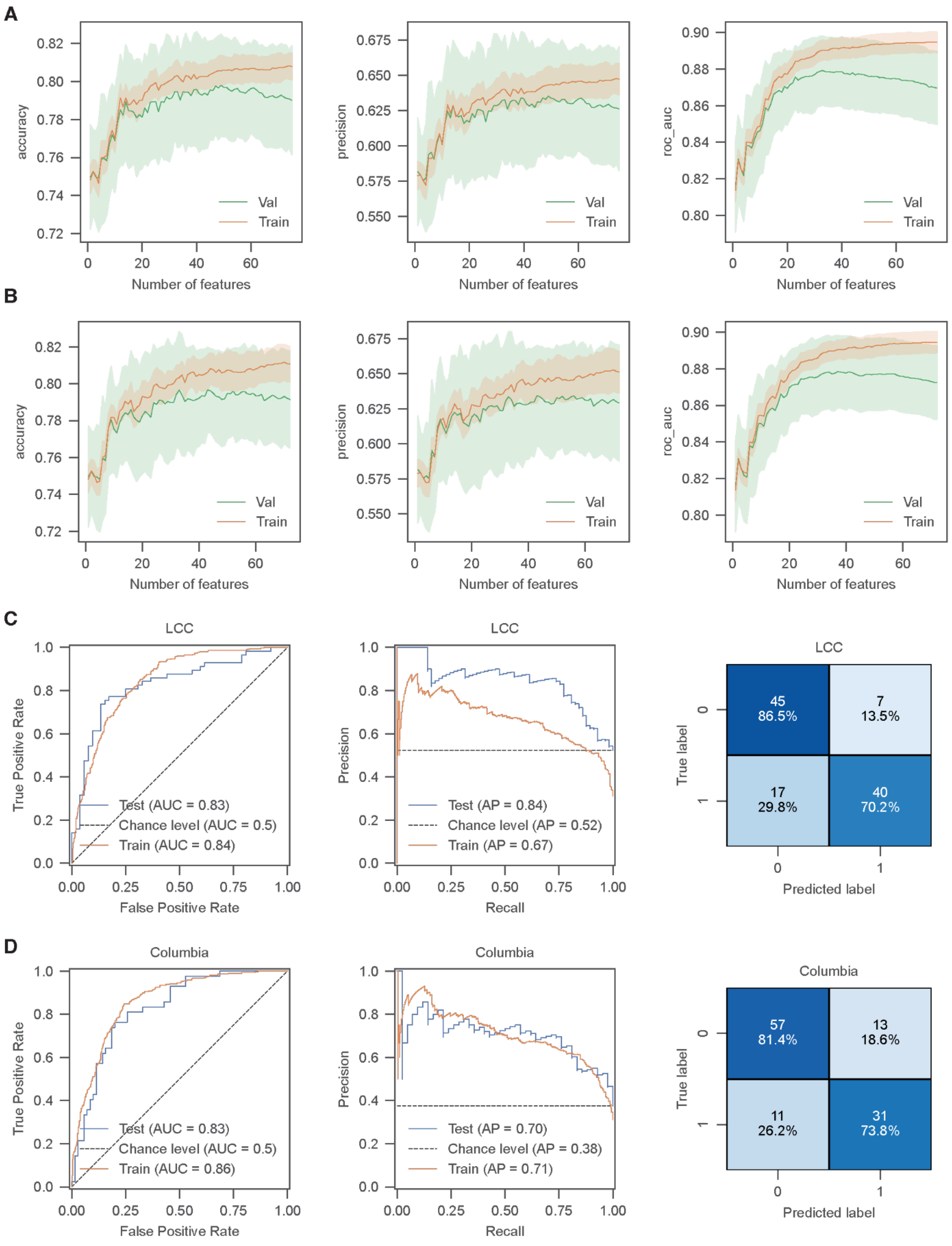

Appendix Figure S6 Cross-cohort prediction of *LRRK2* mutation status

A-B. Evaluation of overfitting with increasing numbers of features as input using accuracy (left), precision (middle), and ROC AUC (right) for prediction of *LRRK2*^{G2019S} or *LRRK2*^{WT}. The evaluation was done on the PPMI urine for training and validation data with (A) testing on LCC and (B) testing on Columbia data. The shaded areas indicate ±1 standard deviation.
C-D. ROC curve (left), Precision-Recall curve (middle), and Confusion Matrix (right) for an SVM classifying *LRRK2*^{G2019S} individuals versus *LRRK2*^{WT}, trained on the entire PPMI data

and tested on LCC using n=10 proteins (C) and Columbia using n=5 proteins (D) datasets. Random performance is represented by the dotted diagonal line.

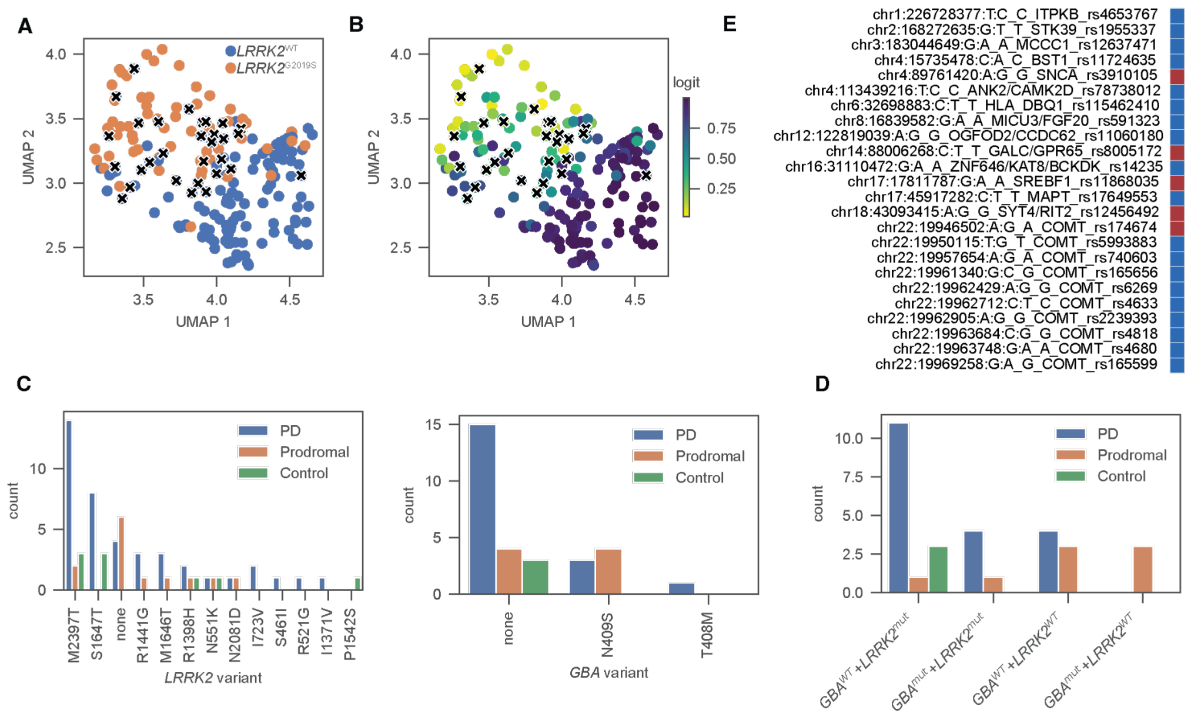

Appendix Figure S7 Genetic risk variants share *LRRK2*^{G2019S} like signature

A. UMAP-reduced dataset consisting of 30 biomarker genes. Colors depict *LRRK2*^{WT} or *LRRK2*^{G2019S} samples. “x” depicts false positive identification.

B. Same as A but coloring depicts predicted logits (probability).

C. Genetic variant status for *LRRK2* (left) or *GBA* (right) of patient with false positive identification.

D. Number of *LRRK2* and *GBA* variants of false positive identification.

E. Genetic risk variants of PD Patient (patient number 3269) without identified *LRRK2* or *GBA* mutation who is falsely classified as *LRRK2*^{G2019S}. Blue depicts heterozygous, red depicts homozygous mutation.

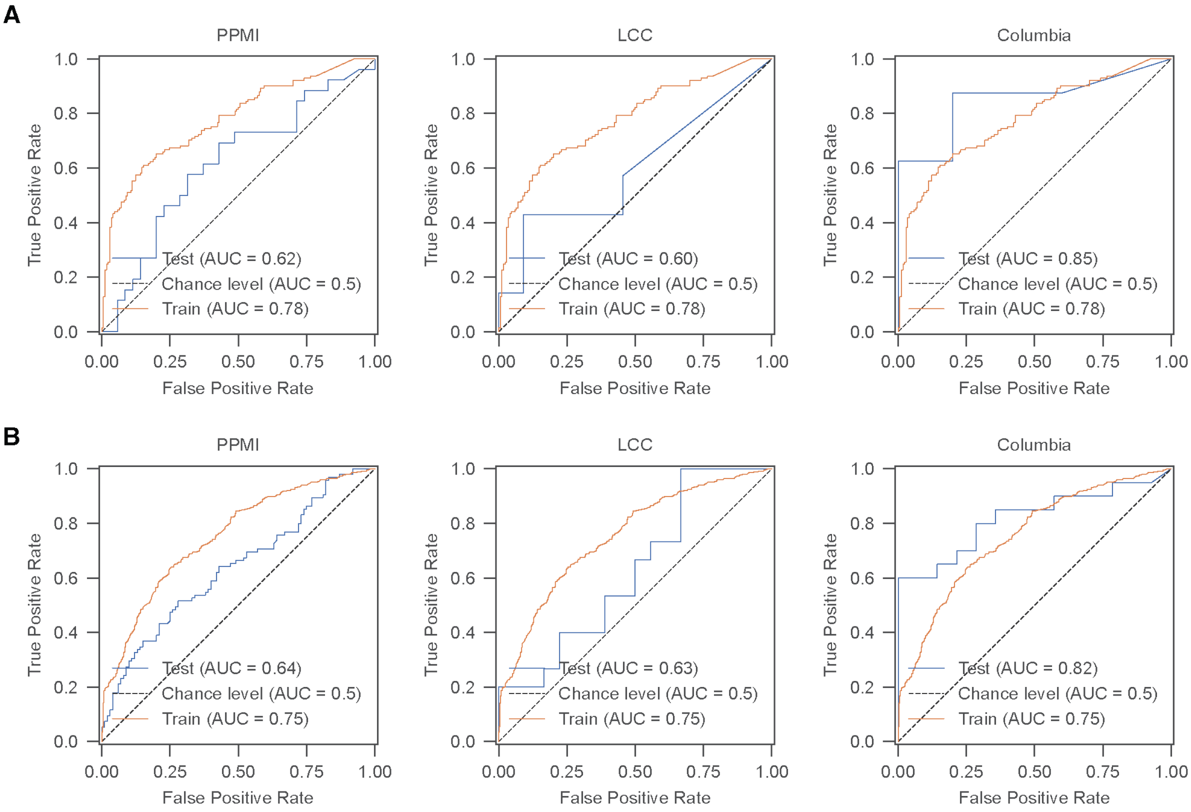

Appendix Figure S8 Machine learning-based classification of *PD*

A. ROC curves for a support vector machine (SVM) classifying PD with *LRRK2*^{G2019S} individuals versus non manifesting controls with *LRRK2*^{G2019S}, trained on combined urine data and tested on PPMI (left), LCC (middle), and Columbia (right) datasets using n=10 proteins. Random performance is shown by the dotted diagonal line.
B. Same as (A) but for PD versus control classification using n=10 proteins.

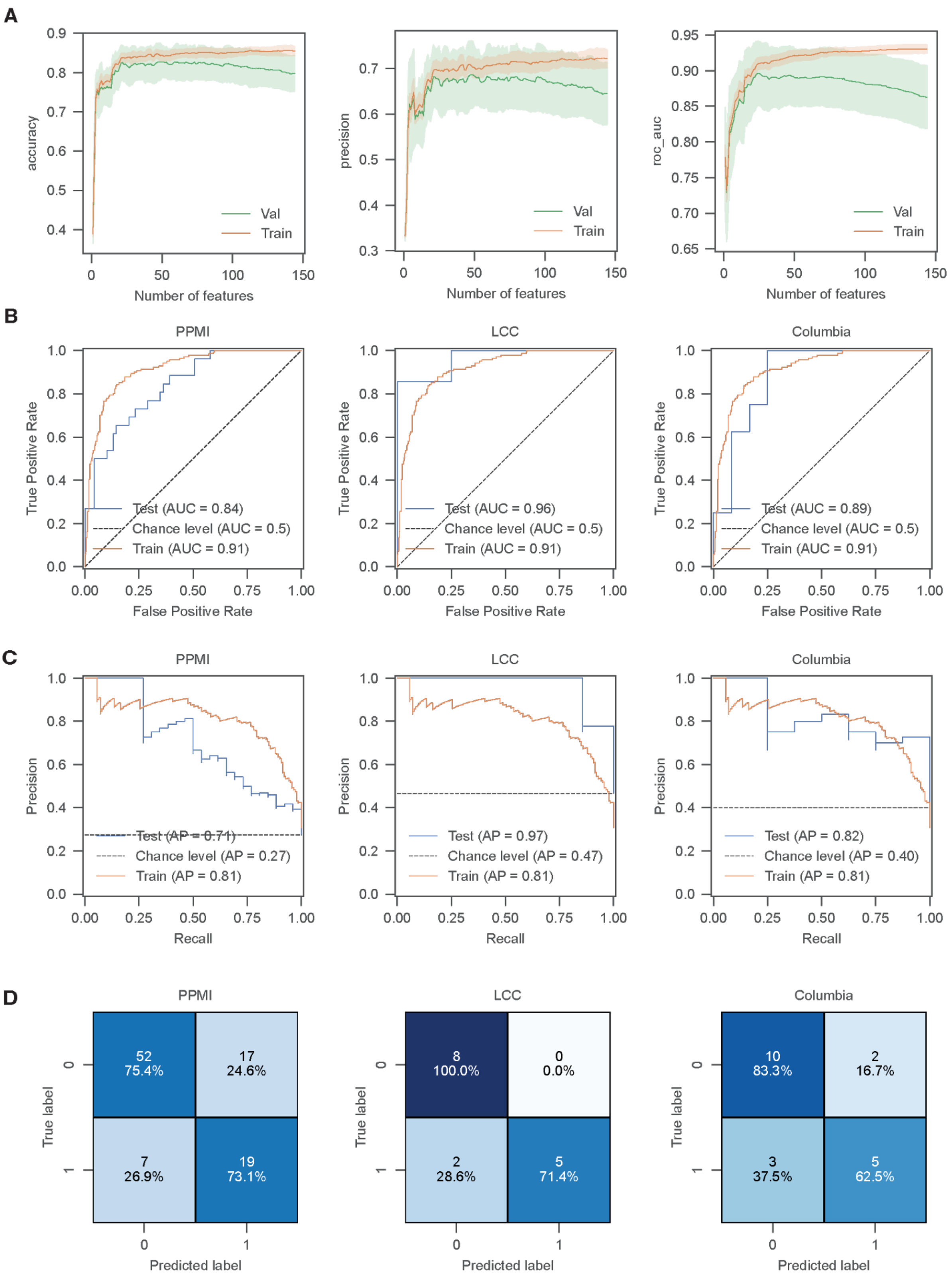

Appendix Figure S9 Machine learning-based prediction of *LRRK2*^{G2019S} status in PD patients

A. Evaluation of overfitting with increasing numbers of features as input using accuracy (left), precision (middle), and ROC AUC (right) for prediction of *LRRK2*^{G2019S} or *LRRK2*^{WT}. The

evaluation was done on the combined (PPMI, LCC & Columbia) urine training and validation data with PD patients only. The shaded areas indicate ±1 standard deviation.

B. ROC curves for a support vector machine (SVM) classifying *LRRK2*^{G2019S} individuals versus *LRRK2*^{WT} in PD patients exclusively, trained on combined urine data and tested on PPMI (left), LCC (middle), and Columbia (right) datasets using n=30 proteins. Random performance is shown by the dotted diagonal line.

C. Same as (B) but model performance was evaluated using precision-recall curve. Average precision (AP) is annotated for the train and test dataset.

D. Confusion matrix for predictions in (B-C) depicting number of true positive predictions in the lower right, true negative predictions in the upper left, false positive predictions in the upper right and false negative predictions in the lower left.

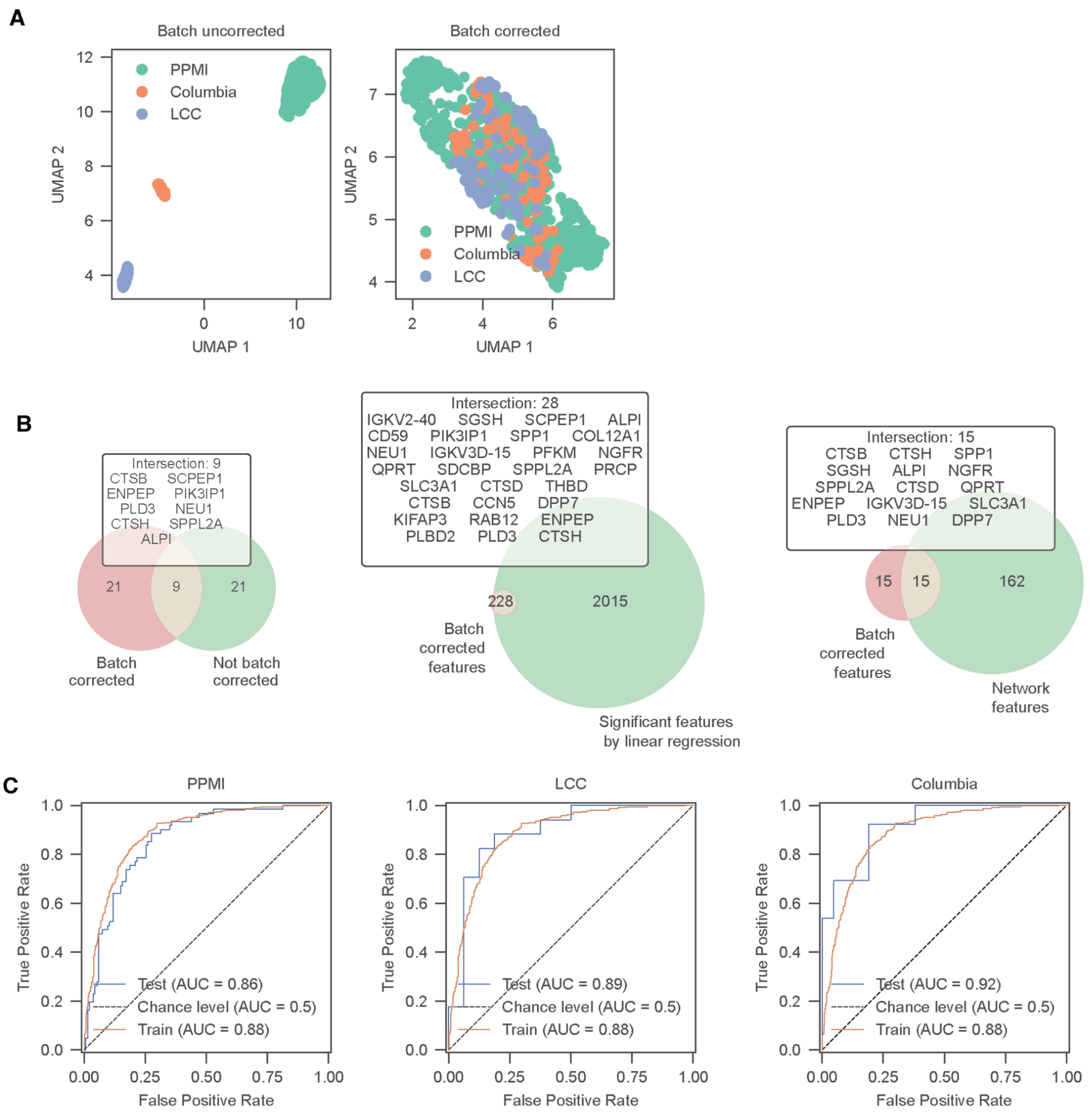

Appendix Figure S10 Batch correction of combined PPMI, LCC and Columbia data

A. UMAP of batch corrected (right) and non-corrected (left) combined dataset (PPMI, LCC and Columbia).
B. Intersection of features selected by model using batch corrected and non-corrected dataset (left), significant features detected by linear regression (middle) and network features (right).
C. ROC curve for an SVM classifying *LRRK2*^{G2019S} individuals versus *LRRK2*^{WT}, trained on the entire batch corrected data and tested on separated and batch corrected PPMI, LCC and Columbia data using n=30 proteins. Random performance is represented by the dotted diagonal line.

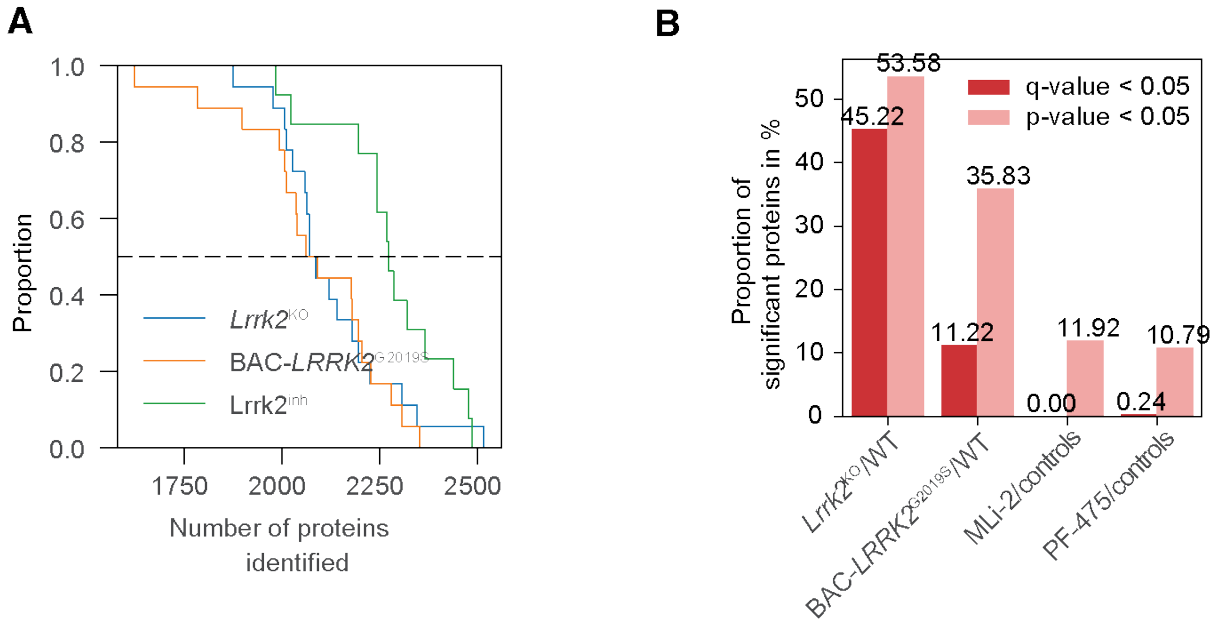

Appendix Figure S11 Peripheral effects of Lrrk2 deficiency, hyperactivation, and inhibition in rat urine

A. Cumulative distribution plot of numbers of identified proteins in the *Lrrk2*^{KO}, BAC-*LRRK2*^{G2019S} and inhibitor dataset. Black dotted line represents the median.
B. Proportion of significant proteins (p-value<5% and q-value<5%) in the urine of BAC-*LRRK2*^{G2019S} and *Lrrk2*^{KO} rats, as well as those treated with the potent LRRK2 inhibitors MLi-2 and PF-475.

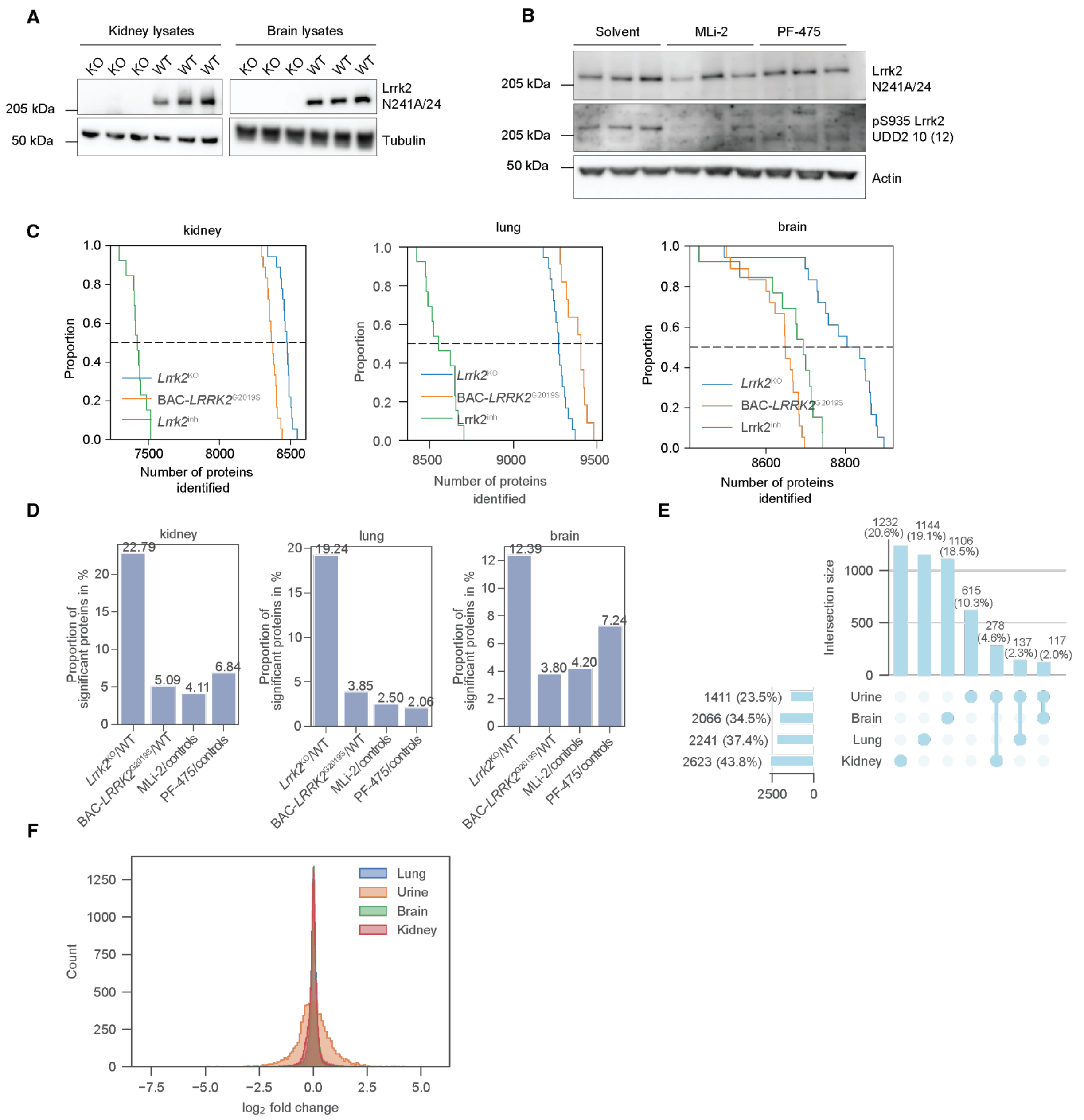

Appendix Figure S12 Effects of Lrrk2 deficiency, hyperactivation, and inhibition in rat kidney, lung, brain and urine

A. Western blots of Lrrk2 and alpha-tubulin (loading control) in kidney and brain lysates from representative n=3 *Lrrk2*^{KO} and n=3 *Lrrk2*^{WT} control rats.
B. Western blots of total and phosphorylated (Serine 935) Lrrk2 and actin (loading control) in kidney lysates of rats 6 hours post-administration with solvent, MLi-2 (2 mg/kg) or PF-475 (30 mg/kg).
C. Cumulative distribution plot of number of identified proteins in the *Lrrk2*^{KO}, BAC-*LRRK2*^{G2019S} and inhibitor kidney (left), lung (middle) and brain (right) dataset. Black dotted line represents the median.
D. Proportion of significant proteins (p-value<5%) in the tissue of BAC-*LRRK2*^{G2019S} and *Lrrk2*^{KO} rats, as well as those treated with the potent LRRK2 inhibitors MLi-2 and PF-475.
E. Intersections of significant proteins (p-value<5%) between urine and tissue samples.
F. Log_2 fold-change distribution between urine and tissue dataset.

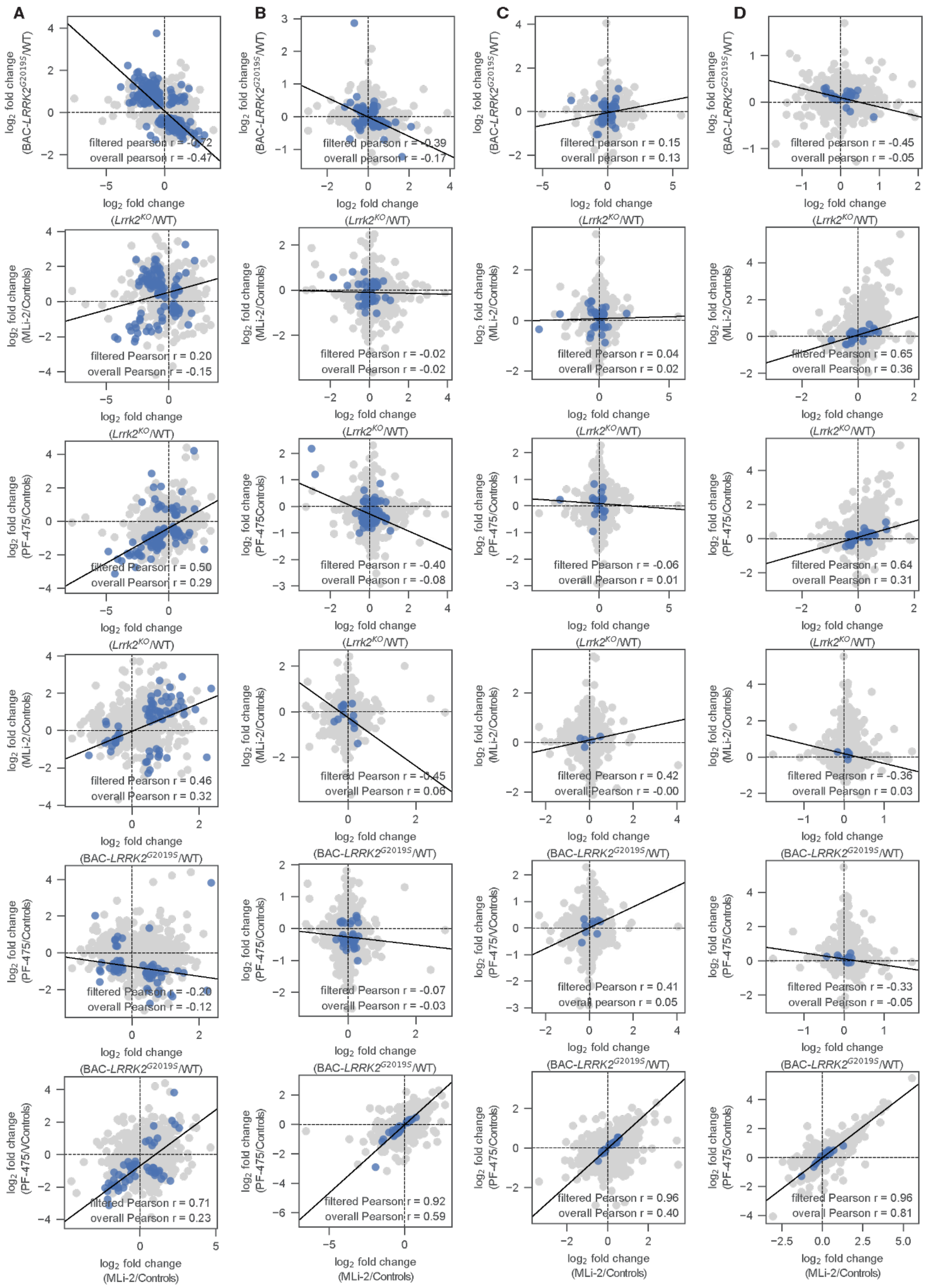

Appendix Figure S13 Correlation analysis in rat urine, kidney, lung and brain

A. Correlation of $\log_2$ fold-changes between *Lrrk2*^{KO} vs. *Lrrk2*^{WT} and BAC-*LRRK2*^{G2019S} vs. *Lrrk2*^{WT} (n=322), *Lrrk2*^{KO} vs. *Lrrk2*^{WT} and MLi-2 vs. Vehicle (n=101), *Lrrk2*^{KO} vs. *Lrrk2*^{WT} and PF-475 vs. Vehicle (n=108), BAC-*LRRK2*^{G2019S} vs. *Lrrk2*^{WT} and MLi-2 vs. Vehicle (n=67), BAC-*LRRK2*^{G2019S} vs. *Lrrk2*^{WT} and PF-475 vs. Vehicle (n=67), MLi-2 vs. Vehicle and PF-475 vs. Vehicle (n=52) in urine with significantly regulated proteins (p-value < 5%) in blue. The overall Pearson correlation coefficient (Pearson r) depicts calculation using all proteins, while filtered indicates the calculation with significant proteins in both comparisons.
B. Same as (A) but for kidney with n=123, n=68, n=120, n=13, n=36, n=54 from top to bottom.
C. Same as (A) but for lung with n=74, n=44, n=22, n=5, n=9, n=29 from top to bottom.
D. Same as (A) but for brain with n=36, n=45, n=77, n=11, n=16, n=92 from top to bottom.

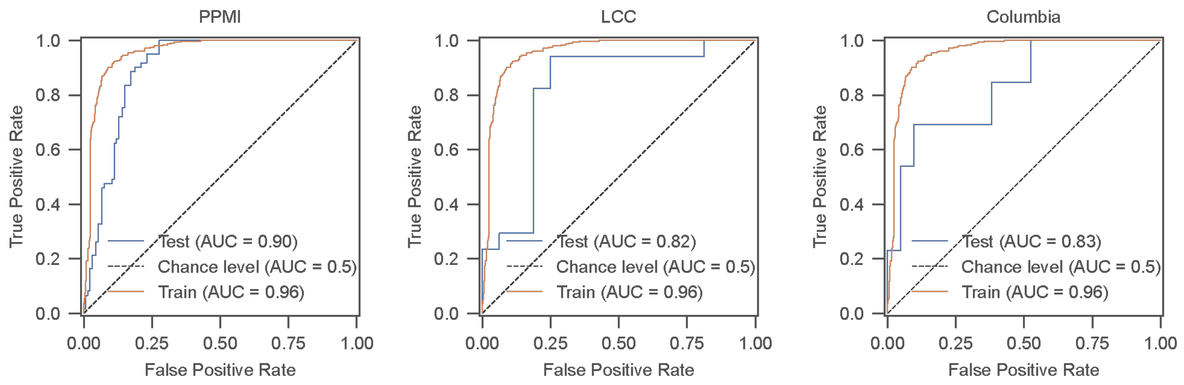

Appendix Figure S14 Model performance of significant rat genes in BAC-*LRRK2*^{G2019S} rats for human *LRRK2*^{G2019S} classification

Receiver operating characteristic (ROC) curves for a support vector machine (SVM) classifying individuals with *LRRK2*^{G2019S} vs. controls using the n=608 significant proteins (p-value<5%) from the rat urine study. The model was trained on combined urine data and tested on cohort-specific datasets: PPMI (left), LCC (middle), and Columbia (right). Random performance is shown by the dotted diagonal line.

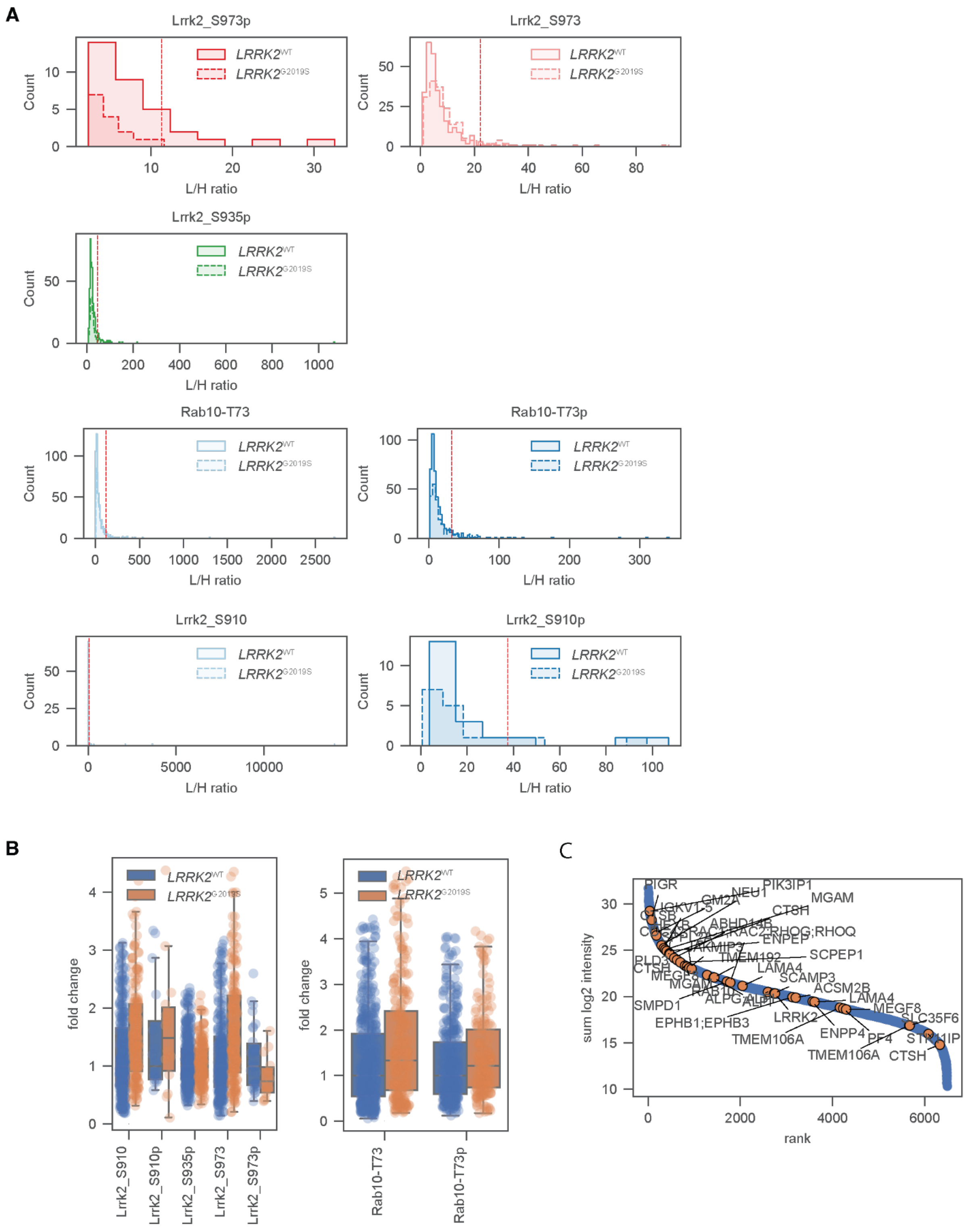

Appendix Figure S15 Targeted proteomic analyses of phosphorylated and unphosphorylated LRRK2 and Rab10 peptides in the PPMI urine dataset

A. Distribution of L/H ratio of LRRK2 and RAB10 peptides. The red dotted line represents +/- 3 standard deviations from the median L/H ratio. Values above red dotted lines were excluded from analysis.

B. Distribution of fold-change (*LRRK2*^{G2019S}/*LRRK2*^{WT}) for LRRK2 and RAB10 peptides. Intensities were normalized to the median intensity of *LRRK2*^{WT} samples.

C. Proteins identified in the PPMI cohort were ranked according to their MS signals. 30 machine learning model features and RAB10 and LRRK2 are annotated in orange in the PPMI dataset.

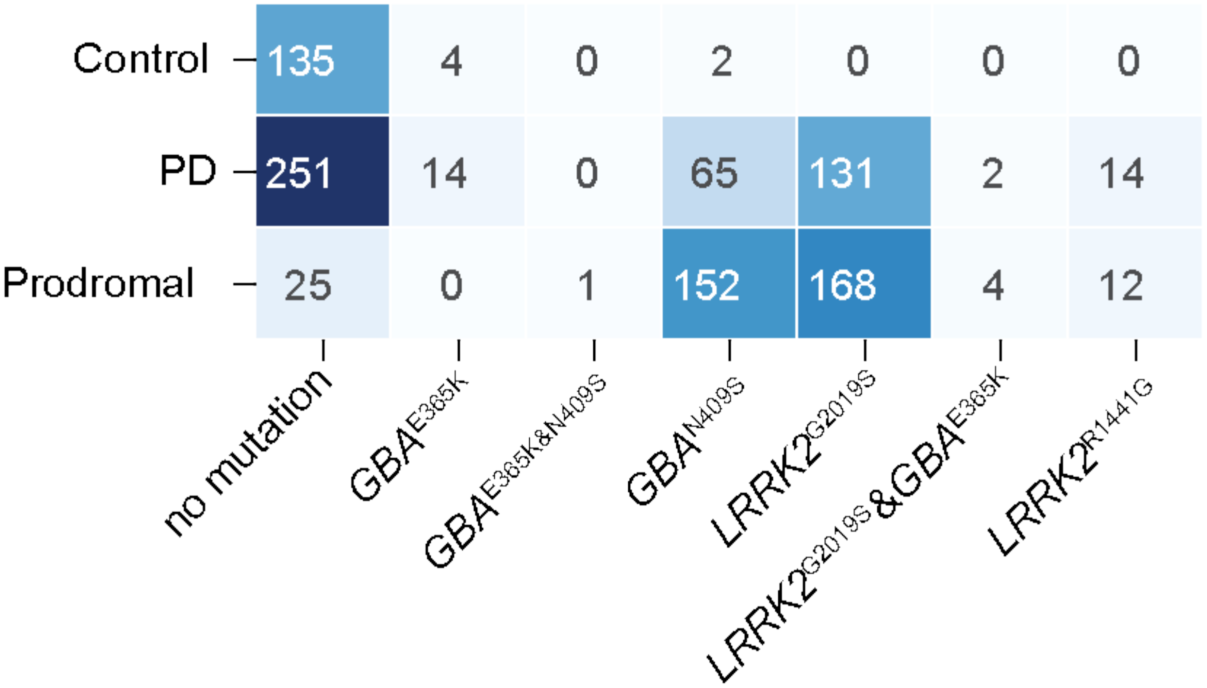

Appendix Figure S16: Distribution of genetic mutations across disease states

A cross-tabulation showing the frequency of genetic variants (columns) across clinical groups (rows) in the PPMI dataset.
