# Supplementary material for: Multi-cohort, cross-species urinary proteomics reveals signatures of LRRK2 dysfunction in Parkinson’s disease
Source: Mol Syst Biol. 2026 Jan 29;22(5):712–37. doi: 10.1038/s44320-026-00190-0 (PMC13144513; doi:10.1038/s44320-026-00190-0)
Supplement: Supplementary file 9 — Expanded View Figures [file 44320_2026_190_MOESM9_ESM.pdf]

Expanded View Figures

Figure EV1. Detecting urinary proteome alterations associated with genetic risk variants in PD.

(**A**) Number of significant proteins for each genetic risk variant based on multiple linear regression analysis on WGS data. Each genetic variant is described by gene name followed by the unique NCBI dbSNP database identifier. (**B**) Pearson correlation coefficient and corresponding $-\log_{10} q$ values of beta-value profile derived from multiple linear regression analysis between *LRRK2*^{G2019S} and other genetic risk variants. (**C**, **D**) Reactome enrichment plot of significant proteins (q value <1% and |beta|>0.2) from linear regression analysis for G2019S and R1441G. The overlap illustrates the proportion of enriched proteins in each term, with the most relevant terms annotated. The combined score represents an aggregated metric calculated during enrichment analysis. (**E**) Volcano plots showing the relationship between $-\log_{10} p$ values and beta values for Prodromal, PD *LRRK2*^{G2019S}, PD, *GBA*^{E365A}, and *GBA*^{N409S} ($n = 6479$). The horizontal dotted line marks the 5% q value cutoff. The vertical dotted line separates the proteins into up- and downregulation. Significantly regulated proteins by *LRRK2*^{G2019S} in both LCC and Columbia are annotated.

 © The Author(s)

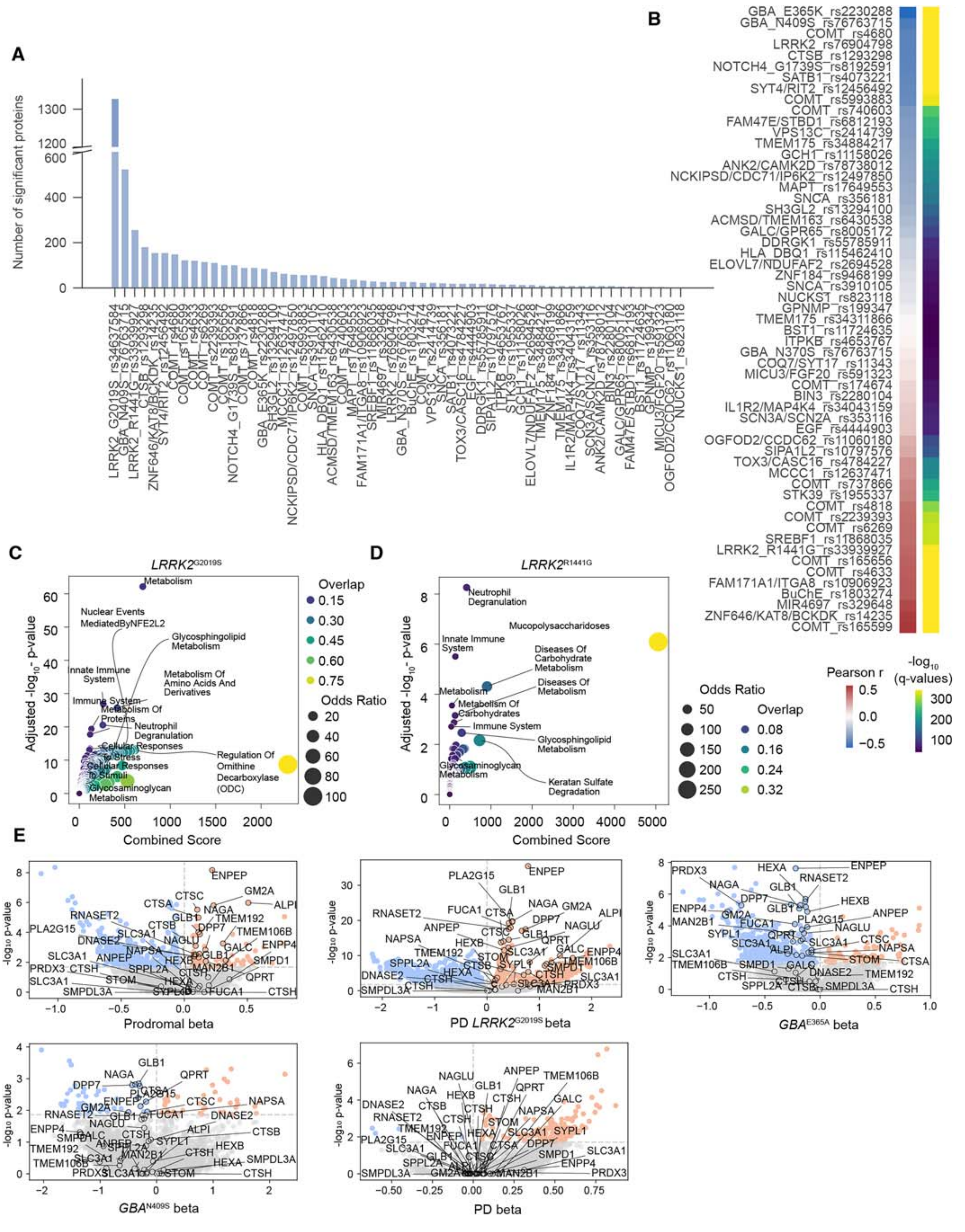
A
Number of significant proteins
B
C
LRRK2G2019S
Adjusted -log10 - p-value
Combined Score
Overlap
Odds Ratio
D
LRRK2R1441G
Pearson r
-log10 (q-values)
E
Prodromal beta
PD LRRK2G2019S beta
GBAE365A beta
GBAN409S beta
PD beta
-log10 p-value

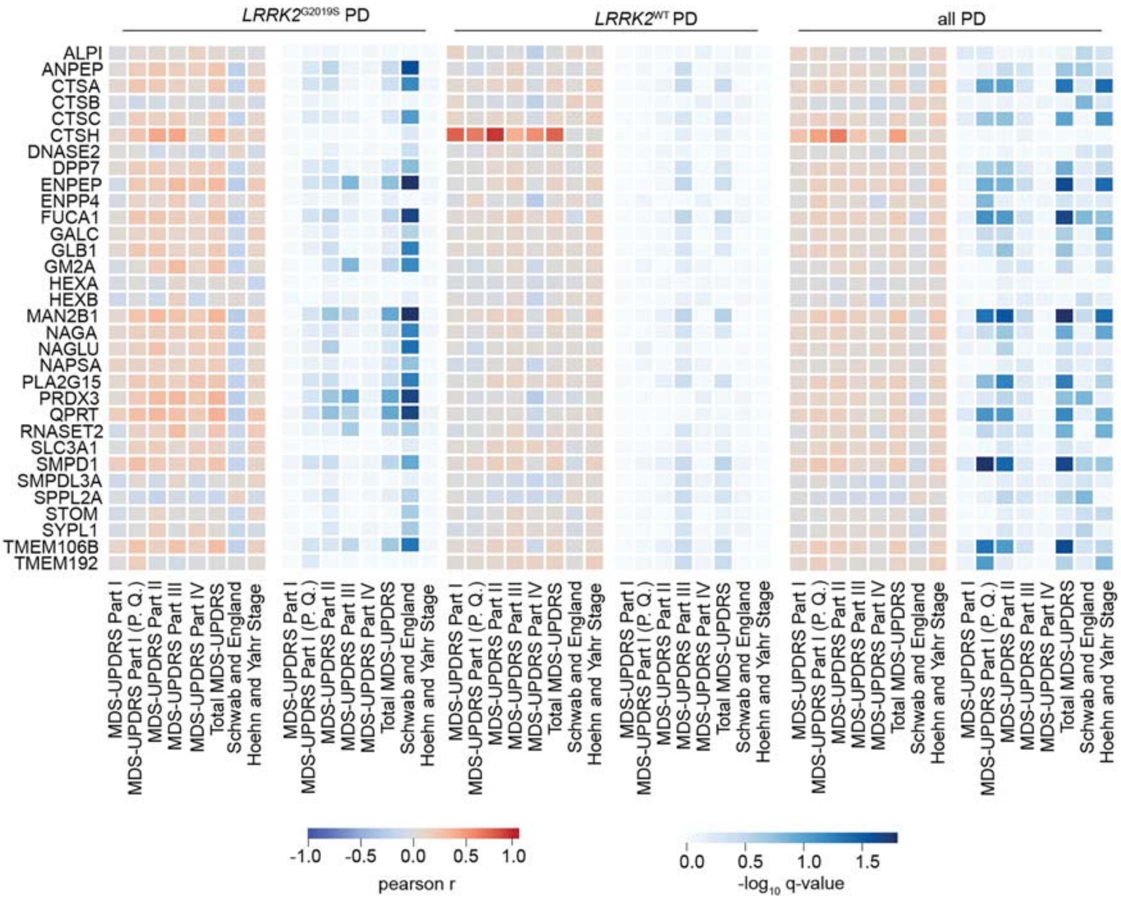

Figure EV2. Correlations between pathogenic LRRK2-regulated proteins from the LCC and Columbia dataset and clinical disease severity scores.

Pearson correlation coefficients and $-\log_{10} q$ values for proteins (found significant in the LCC and Columbia cohort, $n = 32$) associated with severity scores in patients with *LRRK2*^{G2019S}, *LRRK2*^{WT} PD and all PD patients.

© The Author(s)

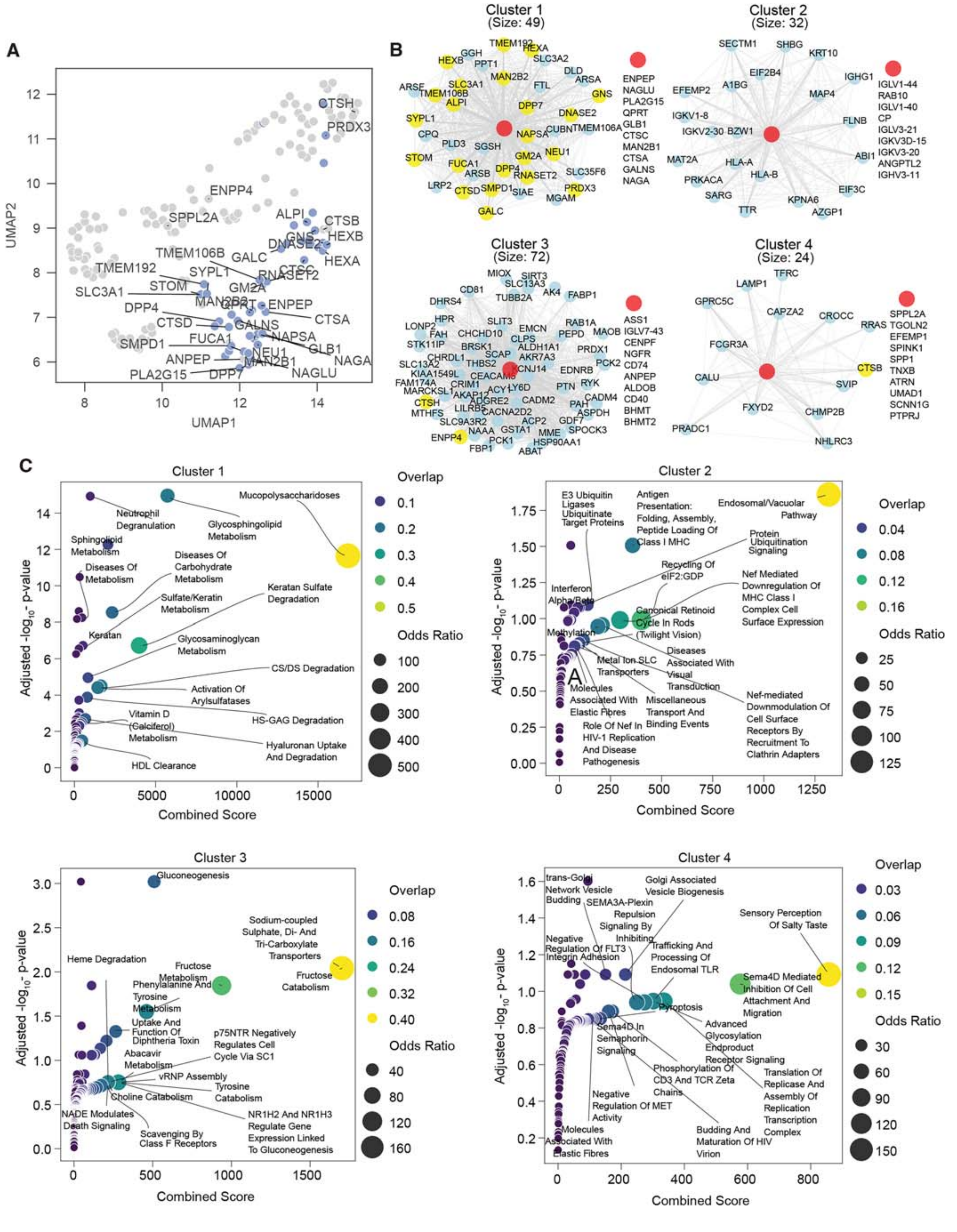
A
UMAP2
UMAP1
CTSH
PRDX3
ENPP4
SPPL2A
ALPI
CTSB
HEXB
HEXA
DNASE2
GNS
TMEM106B
GALC
TMEM192
SYPL1
RNASET2
CTSC
SLC3A1
STOM
GM2A
DPP4
ENPEP
CTSA
CTSD
GALNS
NAPSA
SMPD1
FUCA1
GLB1
NEU1
NAGA
ANPEP
MAN2B1
PLA2G15
DPP7
NAGLU
B
Cluster 1
(Size: 49)
ENPEP
NAGLU
PLA2G15
QPRT
GLB1
CTSC
MAN2B1
CTSA
GALNS
NAGA
Cluster 2
(Size: 32)
IGLV1-44
RAB10
IGLV1-40
CP
IGLV3-21
IGKV3D-15
IGKV3-20
ANGPTL2
IGHV3-11
Cluster 3
(Size: 72)
ASS1
IGLV7-43
CENPF
NGFR
CD74
ANPEP
ALDOB
CD40
BHMT
BHMT2
Cluster 4
(Size: 24)
SPPL2A
TGOLN2
EFEMP1
SPINK1
SPP1
TNXB
ATRN
UMAD1
SCNN1G
PTPRJ
C
Cluster 1
Adjusted -log10- p-value
Combined Score
Overlap
Odds Ratio
Mucopolysaccharidoses
Neutrophil Degranulation
Glycosphingolipid Metabolism
Sphingolipid Metabolism
Diseases Of Carbohydrate Metabolism
Diseases Of Metabolism
Keratan Sulfate Degradation
Sulfate/Keratin Metabolism
Keratan
Glycosaminoglycan Metabolism
CS/DS Degradation
Activation Of Arylsulfatases
HS-GAG Degradation
Vitamin D (Calciferol) Metabolism
Hyaluronan Uptake And Degradation
HDL Clearance
Cluster 2
E3 Ubiquitin Ligases Ubiquitinate Target Proteins
Antigen Presentation: Folding, Assembly, Peptide Loading Of Class I MHC
Endosomal/Vacuolar Pathway
Protein Ubiquitination Signaling
Recycling Of eIF2:GDP
Nef Mediated Downregulation Of MHC Class I Complex Cell Surface Expression
Interferon Alpha/Beta
Canonical Retinoid Cycle In Rods (Twilight Vision)
Methylation
Diseases Associated With Visual Transduction
Metal Ion SLC Transporters
Molecules Associated With Elastic Fibres
Miscellaneous Transport And Binding Events
Nef-mediated Downmodulation Of Cell Surface Receptors By Recruitment To Clathrin Adapters
Role Of Nef In HIV-1 Replication And Disease Pathogenesis
Cluster 3
Gluconeogenesis
Sodium-coupled Sulphate, Di- And Tri-Carboxylate Transporters
Heme Degradation
Fructose Metabolism
Phenylalanine And Tyrosine Metabolism
Fructose Catabolism
Uptake And Function Of Diphtheria Toxin
p75NTR Negatively Regulates Cell Cycle Via SC1
Abacavir Metabolism
vRNP Assembly
Tyrosine Catabolism
Choline Catabolism
NADE Modulates Death Signaling
Scavenging By Class F Receptors
NR1H2 And NR1H3 Regulate Gene Expression Linked To Gluconeogenesis
Cluster 4
trans-Golgi Network Vesicle Budding
Golgi Associated Vesicle Biogenesis
SEMA3A-Plexin Repulsion Signaling By Inhibiting Integrin Adhesion
Negative Regulation Of FLT3
Trafficking And Processing Of Endosomal TLR
Sensory Perception Of Salty Taste
Sema4D Mediated Inhibition Of Cell Attachment And Migration
Pyroptosis
Sema4D In Semaphorin Signaling
Advanced Glycosylation Endproduct Receptor Signaling
Phosphorylation Of CD3 And TCR Zeta Chains
Translation Of Replicase And Assembly Of Replication Transcription Complex
Negative Regulation Of MET Activity
Molecules Associated With Elastic Fibres
Budding And Maturation Of HIV Virion

Figure EV3. Network analysis of pathogenic LRRK2-regulated proteins.

(**A**) UMAP of overlapping pathogenic LRRK2-regulated proteins across three cohorts ($n = 177$), with the significant proteins in LCC and Columbia annotated. (**B**) Clusters from the network of overlapping pathogenic LRRK2-regulated proteins, with yellow nodes for the significant proteins and red nodes for proteins with the highest degree centrality. Clusters were generated using the Louvain clustering algorithm. (**C**) Reactome enrichment plots for clusters 1–4, with overlap indicating the proportion of enriched proteins in each term. The most relevant terms were annotated. The overlap illustrates the proportion of enriched proteins in each term. The combined score represents an aggregated metric calculated during enrichment analysis.

 © The Author(s)

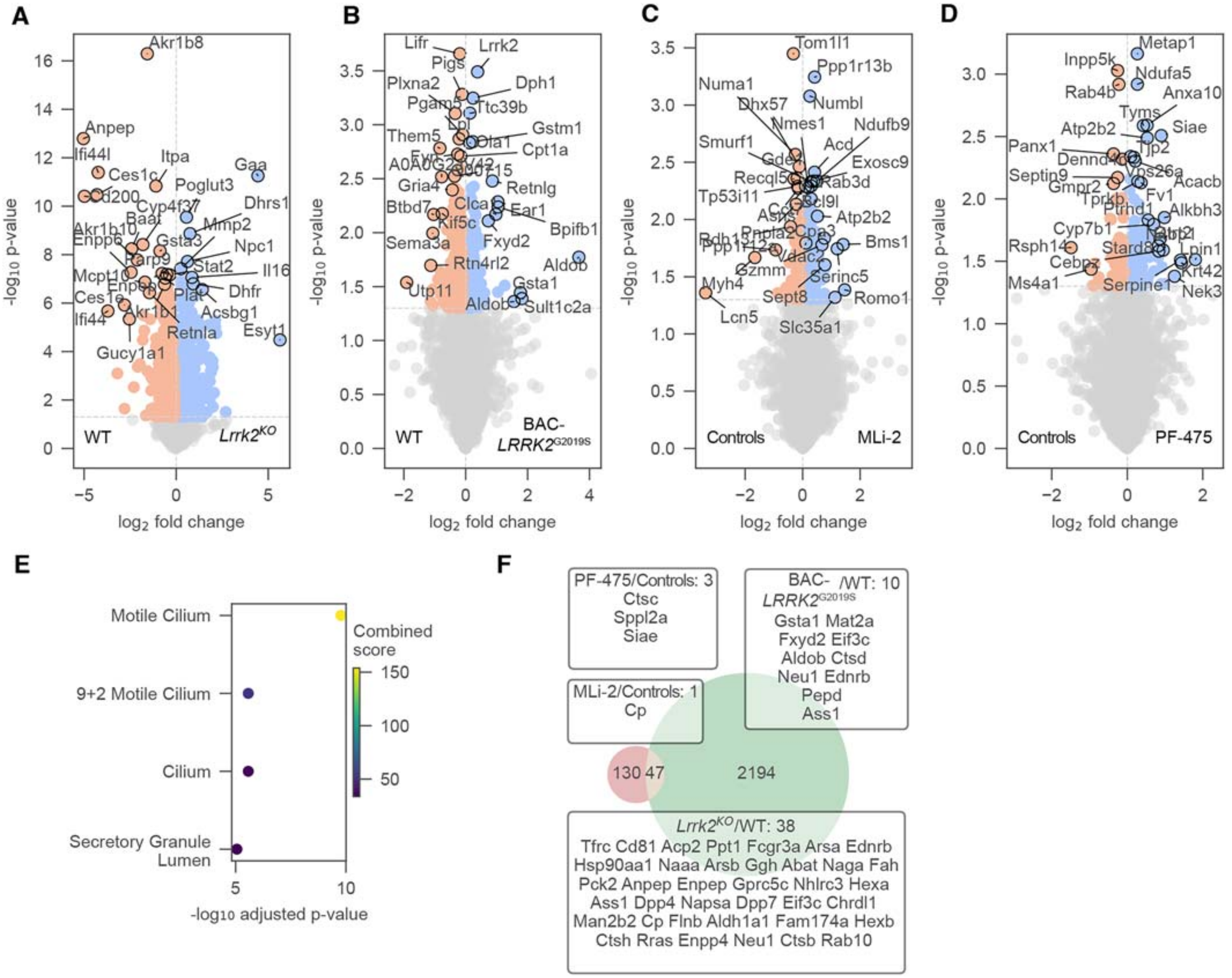

Figure EV4. Detecting proteome alterations associated with Lrrk2 loss, hyperactivation (G2019S), and kinase inhibition in rat lung.

(**A–D**) Volcano plots comparing the lung proteomes of *Lrrk2*^{KO} vs. *Lrrk2*^{WT} rats ($n = 8979$) (**A**), BAC-*LRRK2*^{G2019S} vs. *Lrrk2*^{WT} rats ($n = 9187$) (**B**), MLi-2 vs. Controls ($n = 8447$) (**C**), and PF-475 vs. Controls ($n = 8447$) (**D**) with dotted lines indicating 5% p value cutoffs. The vertical dotted line separates the proteins in up- and downregulation. Most relevant proteins are annotated. (**E**) Enrichment analysis of the GO term "cellular component". Enrichment was performed on significant proteins (p value <5%) in at least two out of four comparisons. The combined score represents an aggregated metric calculated during enrichment analysis. (**F**) Venn diagram of the union of significant proteins (p value <5%) in *Lrrk2*^{KO} vs. *Lrrk2*^{WT} or BAC-*LRRK2*^{G2019S} vs. *Lrrk2*^{WT} or MLi-2 vs. Controls or PF-475 vs. Controls (right) and network genes from the human study (left). Intersecting proteins are annotated in a box with their respective comparison.

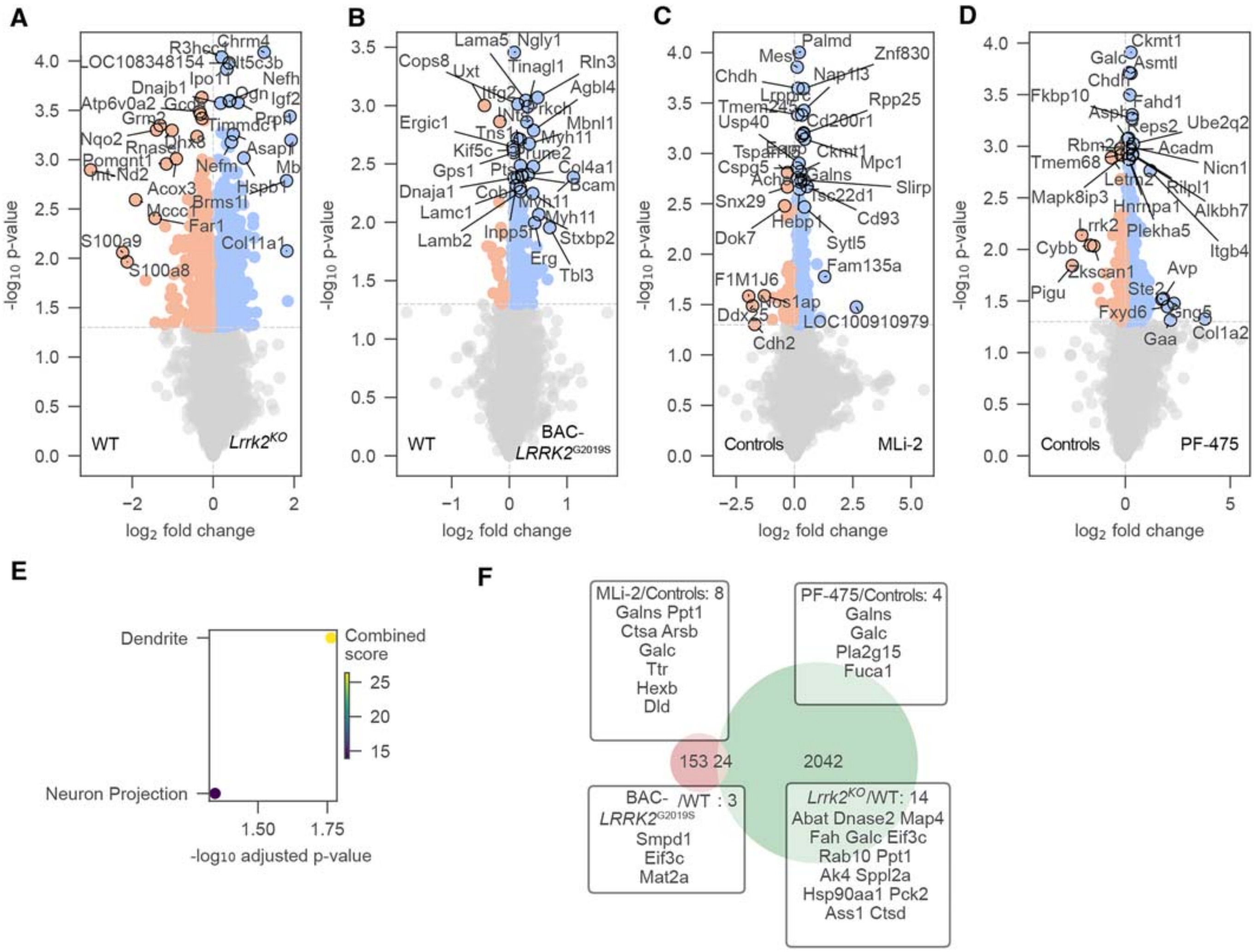

Figure EV5. Detecting proteome alterations associated with Lrrk2 loss, hyperactivation (G2019S), and kinase inhibition in rat brain.

(**A–D**) Volcano plots comparing the brain proteomes of *Lrrk2*[KO] vs. *Lrrk2*[WT] rats ($n = 8499$) (**A**) and BAC-*LRRK2*[G2019S] vs. *Lrrk2*[WT] rats ($n = 8314$) (**B**), MLi-2 vs. Controls ($n = 8639$) (**C**), and PF-475 vs. Controls ($n = 8639$) (**D**) with dotted lines indicating 5% *p* value cutoffs. The vertical dotted line separates the proteins in up- and down-regulation. Most relevant proteins are annotated. (**E**) Enrichment analysis of the GO term "cellular component". Enrichment was performed on significant proteins (*p* value <5%) in at least two comparisons. The combined score represents an aggregated metric calculated during enrichment analysis. (**F**) Venn diagram of the union of significant proteins (*p* value <5%) in *Lrrk2*[KO] vs. *Lrrk2*[WT] or BAC-*LRRK2*[G2019S] vs. *Lrrk2*[WT] or MLi-2 vs. Controls or PF-475 vs. Controls (right) and network genes from the human study (left). Intersecting proteins are annotated in a box with their respective comparison.

© The Author(s)
